# The Value of Character-Based Judgement in the Professional Domain

**DOI:** 10.1007/s10551-019-04269-7

**Published:** 2019-09-05

**Authors:** James Arthur, Stephen R. Earl, Aidan P. Thompson, Joseph W. Ward

**Affiliations:** School of Education, University of Birmingham, Birmingham, UK

**Keywords:** Character-judgement, Character profiles, Professional purpose

## Abstract

Dimensions of character are often overlooked in professional practice at the expense of the development of technical competence and operational efficiency. Drawing on philosophical accounts of virtue ethics and positive psychology, the present work attempts to elevate the role of ‘good’ character in the professional domain. A ‘good’ professional is ideally one that exemplifies dimensions of character informed by sound judgement. A total of 2340 professionals, from five discrete professions, were profiled based on their valuation of qualities pertaining to character and judgement. Profile differences were subsequently examined in the self-reported experience of professional purpose towards a wider societal ‘good’. Analysis of covariance, controlling for stage of career, revealed that professionals valuing character reported higher professional purpose than those overweighting the importance of judgement or valuing neither character nor judgement, *F*(3, 2054) = 7.92, *p* < .001. No differences were found between the two groups valuing character, irrespective of whether judgement was valued simultaneously. This profiling analysis of entry-level and in-service professionals, based on their holistic character composition, paves the way for fresh philosophical discussion regarding what constitutes a ‘good’ professional and the interplay between character and judgement. The empirical findings may be of substantive value in helping to recognise how the dimensions of character and judgement may impact upon practitioners’ professional purpose.

## Introduction

The professions continue to occupy a unique and privileged place in the public eye. They are relied upon for moral probity, diligence, fairness and resolve. Professionals are expected to exercise personal morals informed by judgement in the interests of their organisation, those they immediately serve (e.g. clients, customers, patients, students) and society at large (Carr et al. 2011). It is perhaps because these occupations are held to such a standard that instances of professional misconduct are often followed by outbreaks of outrage, leading to heightened levels of public mistrust towards the professions (e.g. Blond et al. 2015). It is commonly held that instances of malpractice are the result of the ethical shortcomings of ‘bad’ individuals (e.g. Dixon-Woods et al. 2011). However, a more nuanced assessment might understand such incidents as failures or errors of judgement deriving from shortfalls in character on the part of practitioners working within challenging professional contexts. Judgements informed by ‘good’ character are essential for effective and purposeful professional practice, yet practitioners’ character is often not given necessary attention by professional regulators (Furlong et al. 2017). The present research takes a holistic view of professionals’ character, adopting a profiling analytical approach to cluster practitioners from diverse professions based on their valuation of character and judgement. The study offers philosophical and practical interpretation of these character-judgement profiles and examines how they may differ in a perceived ‘good’ purpose for their professional work.

### The Importance of Professionals’ Character

The ‘good’ professional, as traditionally conceived, will have developed the technical competencies for their respective field adjoined with excellences of character required for ethical and systematic deliberation (Carr 2018; Sturm et al. 2017). Senior directors and executives consistently perceive character to play an important role in professional organisations, yet indicate that character is rarely given precedence in organisational cultures, professional training and recruitment processes (e.g. Seijts et al. 2015, 2019). Such discussions are set within a wider context of constraining socio-economic initiatives focused on efficiencies, budget cuts and new management practices, which have had a substantial impact on many professional contexts (see Evetts 2009; Lewis et al. 2017). Within this prevailing culture of auditing and performance metrics, organisations have tended to focus more narrowly on developing the technical efficiencies of practitioners (e.g. Crossan et al. 2013, 2017). Philosopher Alasdair MacIntyre (1981) referred to these technical competencies as the external goods of professional practice, or ‘goods of effectiveness’, which are essential for professionals to be able to succeed in their role, demonstrate proficiency and garner outputs (e.g. financial gain, material goods, or service). However, MacIntyre cautioned against individuals, and wider institutions, becoming overly focused on these external goods at the expense of qualities of character aligned with an achievement of the wider ‘good’. He maintained that precedence should to be given to internal qualities of character, or ‘goods of excellence’, which are necessary for practitioners to be accountable and to think for themselves with humanity and integrity (Beadle and Moore 2011). Indeed, breaches in ethical conduct will rarely be a consequence of technical ineptitude but often grounded upon character-void judgements (Seijts et al. 2017).

Devoting further attention to the character-judgement balance is particularly important within the professional realm as active professionals are responsible for decisions and actions that can have substantial consequences for other individuals and society at large (Sama and Shoaf 2008). Be it within the public, private or not-for-profit sectors, operations within the professional domain have implications for the healthcare, education, social and economic functioning of communities. Endorsing character-informed judgements will better equip practitioners to deliberate over potential actions, problem solve and make conclusive decisions when responding to the unpredictable realities of daily professional life (Evetts 2009; Grossmann 2017). At a corporate level, managerial CEOs decisions that are informed by dimensions of character typically correspond with higher levels of operational performance and decision-making (e.g. Kiel 2015; Sosik et al. 2012). Furthermore, professionals informed by character should be more conscientious of the wider societal good of their work, rather than being directed by self-serving or material objectives which could lead to unethical consequences (Moore 2015, 2017). Given the scarcity of empirical research concerning active professionals’ character, the present study attempts to elevate this dimension, as well as highlight the substantive worth for organisations and regulators to facilitate ‘good’ character within their workforce.

### Character in the Professional Sphere

In the broadest sense, character encompasses positive cognitive, emotional and behavioural habits that guide and motivate human excellence (Kristjánsson 2016). Within the field of moral philosophy, many accounts of character in the professional realm are rooted in the notion of virtue (Moore 2017). Virtues form the centrepiece of an Aristotelian perspective of ‘good’ character, reflecting positive and intrinsic qualities that are both constitutive of and conducive to human excellence (Aristotle 2009, p. 5 [1095a17–21]). Collectively, virtues reflect contextually appropriate traits and values—such as honesty, compassion and perseverance—which become habitually ingrained through deliberate and repetitive practice, predisposing practitioners to behave based on ethically sound habits (Pawar et al. 2017). Although character is an inter-individual attribute, practitioners’ character dispositions, and the corresponding behaviours, can be influenced by the extent the professional context promotes or thwarts ‘good’ character (e.g. Annas 2009). For instance, professional environments that expose practitioners to intense financial and performative pressures may potentially corrode practitioners’ focus on elements of ‘good’ character (Furlong et al. 2017). Gaining greater insights into practitioners’ personal valuations of character may offer a foundation for understanding why some practitioners develop an inherent tendency to excel in their professional service and wider personal life (see Beadle and Moore 2011).

Philosophical accounts of virtue are often posited as distinct from the rule and code-based moral theories of deontology (i.e. Kantian ethics) or the consequentialist focus of utilitarianism (Slote 2010). However, several philosophers have suggested that the rigid distinctions often asserted between these three ethical approaches have been exaggerated, identifying the important, if diminished, role of virtue-led deliberation and judgement in duty and utility-based theoretical accounts (Carr 1999, p. 42; Nussbaum 1999). Specified rules and regulations may not adequately cover responses to all professional situations and often bind practitioners to adhere to prescribed practices (see Banks 2007; Jamal and Bowie 1995). In situations where codes of conduct are ambiguous, practitioners may be at risk of potential episodes of unvirtuous practice if their action does not emanate from character-led autonomous deliberation and reasoning in the given situation (Kristjánsson 2015). While it is important not to disregard the importance of principles, rules and regulations in shaping professionals’ deliberations at work, the contribution of a virtue ethical approach to character is to highlight ways in which professionals draw on qualities of character to make ethically appropriate judgements that are sensitive to the professional context in which they find themselves (Carr et al. 2011, pp. 3–4).

Empirical work concerning character and virtue has typically relied upon the constructs and instruments proposed within positive psychology, focusing in particular on the assessment of individual character strengths^1^ (Peterson and Seligman 2004). This work was intended to operationalise and elucidate the nature of individuals’ self-identified character as it pertained to environments and institutions (Seligman and Csikszentmihalyi 2000). Specifically, 24 strengths of character were put forward which reflect behavioural dispositions of six umbrella virtues: wisdom, courage, humanity, justice, temperance and transcendence. Dispositions of these virtues have shown positive links with greater job satisfaction, work commitment and professional productivity (e.g. Gander et al. 2012; Harzer and Ruch 2013, 2014; Littman-Ovadia and Steger 2010). Moving away from these umbrella virtues, other studies have explored dimensions of character pertinent to organisations (e.g. Bright et al. 2006; Cameron et al. 2004). This work revealed that professional organisations typically perform better and are more sustainable in regard to financial margins, innovative ideas and client/customer service when their practitioners demonstrate virtuous behaviours associated with dispositions of ‘good’ character (e.g. compassion, integrity, trust; Cameron et al. 2004). Practitioners of ‘good’ character are more likely to work collaboratively with colleagues, practice with greater accountability for their decisions and persevere with integrity in their work. This evidence highlights the valuable role that practitioners’ character can have on meaningful and efficient professional practice.

Essential for one to endorse and demonstrate ‘good’ character is an ability to use well-informed judgement (e.g. Darnell et al. 2019; Kotzee et al. 2016; Seijts et al. 2019). Aristotle refers to the overarching meta-virtue known as *phronesis*, or practical wisdom (Aristotle 2009, pp. 106–107 [1140a24–1140b35]), which serves as a moral integrator to critically evaluate and ‘deliberate finely’ about the relative weight of competing virtues (e.g. considerateness versus honesty). Through systematic reasoning, *phronesis* serves to prevent distinct virtues being employed in excess or deficiency which transforms them into vices when operationalised (Schwartz and Sharpe 2010). For example, practitioners that fail to apply judgement in a situation may be at risk of applying courage without the quality of temperance or prudence which could lead to reckless professional conduct. Likewise, a professional that inadequately determines a situation to require humanity but not determination or perseverance may act with indecision and insufficiency. Practical wisdom (i.e. character-based judgement) is emblematic of ‘good’ character and results in practitioners being open-minded, recognising the true variety of circumstances and situations, and being thoughtful and decisive in the action they take (Kristjánsson 2015). Practitioners of practical wisdom will be able to draw upon ‘good’ character in a medial way at various points during their practice while being more attuned to the implications of various possible responses to professional situations. As a consequence, these practitioners will be able to determine when it is appropriate to be compassionate to others, when it may be better to be prudent in responding to a situation, or when decisive action may be required.

The notion of ‘practical wisdom’ is central to MacIntyre’s (1981) teleological account of character-informed judgement within, but not exclusive to, distinct communities or organisations of practice (see Beadle and Moore 2006). MacIntyre’s view of practical wisdom is broader than Aristotle’s and encompasses the adjudication of all professional situations even if there are no clear ethical or moral implications, but will nevertheless include ethical action when the situation requires. Practitioners that employ judgement not informed by qualities of character may have a propensity to utilise judgement in a more instrumental manner inspired by self-serving and ego-driven motives. In such cases, judgement might facilitate practices which are not underpinned by practical wisdom (i.e. *phronesis*), instead expressing similarities with what Aristotle considered to be mere ‘*cleverness*’ (Aristotle 2009, [1144a23–31]). MacIntyre (1981) cautions that these professionals may have a rational tendency to use judgement to achieve personal ends which are devoid of the internal goods of character needed to fulfil the wider ethical interests of the profession and those they serve. Imbalances between character and judgement would seemingly bring about blind spots that cause practitioners to lose sight of the true purpose they serve and impede effective decision-making. Such an imbalance may consequently result in misguided professional action and incidents of professional malpractice.

Aristotle’s grounding of virtuous character-judgement (i.e. *phronesis*) is not only concerned with good action as a symptomatic end state but is realised through the internalisation of ‘good’ character into one’s psyche (Annas 2009). For instance, although multiple professionals may seem to act similarly from an external perspective, the truly ‘good’ practitioner will have an internalised value of well-informed character which helps regulate cognitive and emotional processes (Darnell et al. 2019; Kristjánsson 2016). Values are central to who individuals are, and may be revealing of practitioners’ true disposition for well-informed character. In accord with the proposed benefit of character-based judgement (e.g. MacIntyre 1981; Seijts et al. 2019), a fully developed character profile is surmised to reflect a concurrent value of character and judgement with neither dimension being given prominence at the expense of the other (Schwartz and Sharpe 2006). In contrast, practitioners that potentially overweigh or underrate the importance of either character or judgement may exemplify imbalanced character profiles.

Empirically profiling professionals upon their individual character composition would be best suited to a person-centred methodology (Howard and Hoffman 2017). Traditionally, studies measuring character in organisations have adopted variable-centred approaches which consider distinct dimensions of character and judgement in isolation (e.g. Andersson et al. 2007; Harzer and Ruch 2015; Waters 2012). In reality, sub-groups of professionals will likely exist that vary in their valuation of character and judgement (see Bergman and Andersson 2010; Morin et al. 2017). Although previous person-centred studies have grouped professionals upon differences in commitment mind-sets (Meyer and Moyin 2016), motivation types (Howard et al. 2016) and environmental supports (e.g. workload, job control and social support; Mäkikangas et al. 2018), no study to the authors’ knowledge has profiled professionals based on the distinct components of character and judgement. The application of such a methodology would allow the synergy between character and judgement to be examined regarding the pattern between dissimilar profiles (i.e. at an inter-individual level) as well as the degree of differentiation within each profile (i.e. at an intra-individual level). Furthermore, identifying professional typologies could have practical implications for the training of professionals as regulators may seek to consider and guide the character composition of both pre- and in-service practitioners.

### Character-based Judgement and Professional Purpose

Embedded within virtue ethical accounts of character is the philosophical notion of a *telos,* or declared purpose (MacIntyre 1981). The logic follows that judgement informed by character, synonymous with *phronesis*, aligns with a greater purpose for one’s activities, work or practice (Aristotle 2009, [1097b20–21]). Although the specific duties, goals and objectives may be unique to different fields, all professionals would be inherently expected to use character-based judgements to exercise moral and social service to others to some degree (Moore 2017, pp. 38–39; also see Colby and Sullivan 2008). True purpose has been stated to reflect an “intention to accomplish something that is at once meaningful to the self and of consequence to the world beyond the self” (Damon et al. 2003, p. 121). Practitioners reporting indicators of ‘good’ character are more likely to view their working role as a ‘*calling’*’—that is, to do meaningful work for the betterment of others and society (e.g. Harzer and Ruch 2012; also see Dik & Duffy 2009; Dik et al. 2015). Furthermore, business organisations’ promotion of character in the workplace has been associated with greater moral attentiveness and increased social responsibility from their practitioners (Dawson 2018)^2^. While these studies do not draw on distinctions of character when infused with judgement, they unearth insights into the empirical links between character and an overarching drive to cultivate organisational and societal thriving towards a common good. Although certain professionals may still report a subjective sense of purpose for their work even when cultivating vice-like qualities, such as greed or recklessness, it is unlikely this purpose will be for the greater good of others but rather for self-serving ends. A ‘vicious’ sense of purpose, such as striving for financial gain through the exploitation of others, or an externally driven purpose, deriving from coercion to work towards someone else’s desires, would not be aligned with a value or cultivation of the internal goods of character (Aristotle 2009, [1166b4–29]). The present research attempts to examine how practitioners that differ in their valuation of character and judgement may vary in their reported experience of professional purpose, with purpose reflecting a volitional and personal commitment to do useful work for the betterment of others and society (Kempster et al. 2011).

## The Present Research and Hypotheses

The principal aim of the present research was to identify distinct profiles of entry-level and established professionals that differ in their personal valuation of character-based judgement. A profile valuing qualities of character and judgement in unison was surmised to reflect a profile that may resemble what Aristotle constitutes as ‘*phronetic virtue’*, that is character infused with judgement (2009, [1142b23–32]). In contrast, practitioners would be grouped into two alternative profiles depending if they valued character at the expense of judgement or valued judgement at the expense of character. A fourth profile would comprise practitioners that seemingly devalue both judgement and character concurrently. The four character profiles were subsequently examined in the extent to which they varied in their perceived sense of professional purpose towards a common ‘good’. Inferring from philosophical links between *phronesis* and a purposeful *telos* (e.g. Aristotle 2009; MacIntyre 1981), it was expected that professionals valuing judgement and character simultaneously would report the highest levels of professional purpose, compared to the other three groups. In accordance with previous evidence (e.g. Dawson 2018; Harzer and Ruch 2012), it was hypothesised that professionals valuing dimensions of character, even with less value placed on judgement, may still report some level of purpose but not to the same degree as a character-judgement profile. In contrast, it was surmised that practitioners who valued judgement at the expense of character would to report lower professional purpose than groups valuing dimensions of character. These practitioners may potentially endorse self-serving motives that do not correspond with the wider societal purpose that professions are expected to serve (Moore 2017). At the opposite extreme, it was hypothesised that professionals valuing neither qualities of character nor judgement would report the lowest experience of professional purpose.

## Methodology

### Participants

A total of 2340 professionals (*M*_age_ = 36.48, SD = 14.33, 60% female, 40% male) participated in the study, deriving from the professions of medicine (*n* = 19%), law (*n* = 25%), teaching (*n* = 12%), business (*n* = 23%) and nursing (*n* = 21%). With regard to stage of career, 49% were entry-level professionals having just completed their course of study or professional training, and 51% were established professionals with at least 5 years of practical experience in their respective field. The ethnic make-up of the cohort was 84% Caucasian, 9% Asian or Chinese, 3% either Black-African or Black-Caribbean, 1% Arabian and 3% reported being multiracial or from other ethnic backgrounds. The participants were predominantly UK nationals (93%), with 7% reporting non-UK based nationality.^3^

### Measures

#### Indicators of Character and Judgement

To tap into professionals’ valuations of character and judgement, participants were asked to rank in hierarchical order their top six most important qualities from a list of 24 character qualities as specified in the Values in Action Inventory of Strengths (VIA-IS; Peterson and Seligman 2004). The 24 specific character qualities were as follows: Appreciation of Beauty, Bravery, Creativity, Curiosity, Fairness, Forgiveness, Gratitude, Honesty, Hope, Humility, Humour, Judgement, Kindness, Leadership, Love, Love of Learning, Perseverance, Perspective, Prudence, Self-Regulation, Social Intelligence, Spirituality, Teamwork and Zest. Participants responded to the statement “*which of the qualities best describe the sort of person you are?*” and rank each quality in descending order. A value of 1 depicted their most valued quality and a score of 6 reflected their sixth most valued. Rankings were reverse-point scored (e.g. a ranking of 1 was assigned a score of 6, a ranking of 2 assigned a score of 5, etc.) and any quality not ranked given a score of 0.

The use of hierarchical rankings forces professionals to discriminate their preference of specific qualities within a given context (Dunn-Rankin et al. 2014). It is important to note that such a method is not strictly aligned with the ontological perspective of character that proposes all dimensions of character to be interconnected (see interdependent nature of character and virtue; Schwartz and Sharpe 2006). A criticism of the notion of character strengths within positive psychology is that it tends to isolate distinct dimensions or qualities of character (Banicki 2014). A professional of ‘good’ character would *ex hypothesi* not prioritise certain dimensions of character at the expense of others but rather synergise all dimensions in a finely regulated balance. The use of a ranking method was intended to tap into professionals’ general character disposition by identifying the character qualities they personally prioritise, rather than directly assess the extent they actually endorse each quality. Indeed, a practitioner may give importance to qualities such as fairness, teamwork or creativity but still simultaneously endorse other dimensions of character to an equal or even greater degree. Conversely, they may place value upon certain qualities but be unable to exercise these qualities due to imposed external demands and a lack of social support within their working environment. With these considerations in mind, the subsequent identified profiles are indicative of professionals’ character values as opposed to the character qualities they exhibit.

#### Professional Purpose

Professionals’ perceptions of their sense of professional purpose were assessed using six positively worded items, adapted from a Europe-wide workplace survey (Eurofound Working Conditions Survey 2010). In line with the definition of professional purpose (e.g. Kempster et al. 2011), these items tapped into professionals’ personal feelings of commitment and engagement towards their work (e.g. “*I am motivated to work to the best of my ability*” and “*I am emotionally involved in my work*”), their perception of doing meaningful work for the betterment of society (e.g. “*I have the feeling of doing useful work to make a social contribution*”), and their sense of volition towards their work (e.g. “*I am able to apply my own ideas in my work*” and “*I am able to influence decisions that are important for my work*”; for wording of all six items see Table 1). Participants read the statement “*Please indicate how often this has been the case in the environment in which you work*” and rated each item on a 5-point scale, ranging from 1 (*Never*) to 5 (*Always*). All six items loaded appropriately onto a professional purpose factor (i.e. > 0.32; Tabachnick and Fidell 2007), according to the Guttman–Kaiser criterion (eigenvalue > 1; Guttman 1954), and demonstrated good internal consistency (*α* = .79). All item loadings, eigenvalues and explained variance are presented in Table 1.

**Table 1:** Confirmatory factor analysis for professional purpose items

	Factor loading
I am motivated to work to the best of my ability	0.68
I am able to apply my own ideas in my work	0.69
I feel ‘at home’ in my workplace	0.71
I have the feeling of doing useful work to make a social contribution	0.76
I am emotionally involved in my work	0.60
I am able to influence decisions that are important for my work	0.75
**Eigenvalue**	**2.94**
**Explained variance**	**49.02%**

Numbers in bold text signify the eigenvalue and percentage variance accounted for by the professional purpose factor

Extraction method: principal component analysis. Eigenvalue > 1

### Procedure

Full ethical approval was obtained from the research team’s university ethics committee. Entry-level professionals were recruited on completion of their university degree or professional training (e.g. Qualified Teacher Status or Legal Practice Courses), whereas established professionals were predominantly recruited through university alumni offices and a range of profession-specific organisations and regulatory bodies. Prior to the study commencing, a hardcopy of the survey was piloted with students studying in each respective profession at the host university to check the clarity of terms and comprehension. All participants were provided with full information regarding the study and gave signed informed consent in duplicate to illustrate their willingness to participate. All participants were instructed that they did not have to complete any question if they did not wish to and had the right to withdraw or modify their contribution prior to data analysis. The survey was completed online, with a hardcopy version available to those who desired it, and took a maximum of 15 min to complete.

### Analytical Approach

In the first instance, preliminary analysis involved calculating the mean scores for practitioners’ valuation of each character quality (see Table 2). The profiling of professionals upon character-judgement foregrounds the gap between the conceptualisation of character and its application within a professional context. Bridging this gap requires both an understanding of the theoretical constructs and how the relevant terminology applies within a professional workplace. One example of this tension pertains to the measurement of judgement. The specific qualities of judgement, perspective, creativity, curiosity and love of learning were originally categorised under the umbrella of term of *wisdom* in the VIA (Peterson and Seligman 2004). These qualities were originally collated to reflect the acquisition and use of knowledge. Subsequent analysis has revised this categorisation, suggesting these virtues more accurately reflect individuals’ cognitive engagement and inquisitiveness with their surrounding environment (McGrath 2015; Shyrack et al. 2010). Although such distinctions diverge slightly from ‘judgement’ being used to deliberate over the means of dimensions of character, the aggregation of these *wisdom* qualities offers a practical method to assess professionals’ value of independent-thought and decision-making which are central components of Aristotle’s portrayal of *phronesis* (Darnell et al. 2019). Based on these aforementioned considerations, a composite variable reflecting judgement was collated using the mean ranking scores for the qualities of judgement, perspective, creativity, curiosity and love of learning.

**Table 2:** Descriptive statistics for ranking scores of character qualities

Character quality	Mean	SD
1. Honesty	2.76	2.44
2. Fairness	2.37	2.33
3. Kindness	1.88	2.32
4. Humour	1.45	1.94
5. Teamwork	1.32	1.90
6. Perseverance	1.18	1.87
7. Judgement	1.04	1.84
8. Leadership	1.01	1.79
9. Love of Learning	0.95	1.75
10. Curiosity	0.90	1.75
11. Social Intelligence	0.82	1.68
12. Creativity	0.73	1.62
13. Perspective	0.67	1.45
14. Love	0.53	1.44
15. Modesty	0.43	1.18
16. Self-Regulation	0.43	1.19
17. Gratitude	0.39	1.16
18. Forgiveness	0.36	1.14
19. Bravery	0.32	1.13
20. Appreciation of Beauty	0.32	1.11
21. Hope	0.32	1.05
22. Spirituality	0.28	1.08
23. Zest	0.19	0.85
24. Prudence	0.19	0.78

Character qualities are positioned in hierarchal order based on mean ranking scores

In regard to other dimensions of character, Crossan et al. (2017) identified issues with specific character qualities and their suitability to organisational domains. For example, the quality of love was found to be problematic for professionals to conceptualise in a work context. Instead, the term compassion was found to be a more professionally appropriate alternative which is analogous to the quality of kindness in the VIA. Qualities such as humour and spirituality were also viewed as contextually irrelevant by professionals and not in sync with the requirements of their day-to-day working culture. The qualities of love and spirituality have consistently been identified within a transcendent or theological dimension of character, along with qualities such as appreciation of beauty, gratitude, hope and forgiveness (e.g. McGrath 2014; Ruch et al. 2010; Singh & Choubisa 2010). While these strengths should not be discounted in light of evidence of their strong associations with individual well-being and life satisfaction (e.g. Littman-Ovadia and Lavy 2012; Park et al. 2004), they may not represent essential elements of character as pertaining to the professional realm. The transcendent qualities of forgiveness and gratitude often simultaneously reflect strengths of emotional care towards others in general life (McGrath 2014; Shryack et al. 2010), and hope is synonymous with future-mindedness associated with greater life satisfaction and meaning (Feldman & Snyder 2005). Conversely, interpersonal care in a professional context may be more concisely encapsulated by qualities in the VIA such as kindness, fairness and social intelligence, whereas work-minded commitment may be better reflected by qualities such a zest and perseverance which are comparable with the character dimension of *drive* in an organisational setting (i.e. passionate engagement towards excellence; Crossan et al. 2017; also see Peterson et al. 2009). Thus, in order to collate a succinct reflection of character relevant within a professional domain, the quality of humour along with the transcendent and theological qualities of spirituality, love, appreciation of beauty, gratitude, forgiveness and hope were excluded from the aggregation of the character variable. The composite character variable, therefore, comprised 12 qualities: bravery, fairness, honesty, kindness, leadership, modesty, perseverance, prudence, self-regulation, social intelligence, teamwork and zest. This character composite was judged to be representative of the dimensions of professional character proposed by Crossan et al. (2017), as well as tapping into integral components of the original VIA (e.g. courage, humanity, justice, temperance) and equivalent component analyses (e.g. interpersonal care, emotional functioning, self-control; see McGrath 2015; Ruch et al. 2010).

Both the character and judgement composite variables were subsequently used as clustering criteria to profile professionals. To reduce the impact of any statistically abnormal deviations from the mean in either clustering variable, univariate outliers (*z*-score values ± 3.29, *p* < 0.001; Tabachnick and Fidell 2001) and multivariate outliers (individuals with high Mahalanobis values) were removed prior to the profiling of professionals. Both clustering variables, as well as the variable of professional purpose, were standardised to enable easier interpretation of profile plots (i.e. values above zero represented results above the sample average, whereas scores below zero reflected results below the sample average; see Meyer and Morin 2016). Chi-square difference tests were conducted to examine the distribution of gender and stage of career across the professional profiles. Univariate analyses of covariance (ANCOVA) were conducted to examine differences in professional purpose between the professional profiles. Significant ANCOVA were followed up by post hoc comparisons to explore specific group differences.

## Results

Mean ranking scores for professionals’ valuation of the 24 character qualities are presented in Table 2. Furthermore, descriptive statistics and bivariate correlations for the aggregated character and judgement variables, as well as professional purpose, are presented in Table 3. Prior to creating the professional profiles, five univariate outliers were removed as they demonstrated extreme deviations from the mean in the judgement variable, along with three multivariate outliers that revealed high Mahalanobis distances when combining character and judgement. These procedures are valuable in person-centred methodologies as abnormalities in the data may result in misrepresentation of the identified profiles. Exclusion of these outliers was conducted cautiously as deviant observations may be representative of distinct subpopulations within a sample (Mäkikangas et al. 2018). Examination of the identified outliers, however, revealed this was not the case as the eight outliers appeared random across multiple professions (business = 5, law = 2 and teaching = 1).

**Table 3:** Descriptive statistics and bivariate correlations for character, professional judgement and professional purpose

Variable	Mean	SD	1	2	3
1. Character	1.07	0.38	–		
2. Judgement	0.74	0.69	− 0.57^*^	–	
3. Professional purpose	3.80	0.61	0.09^*^	− 0.02	**–**

*p < .001

### Character-Judgement Profiles

**Fig. 1:**
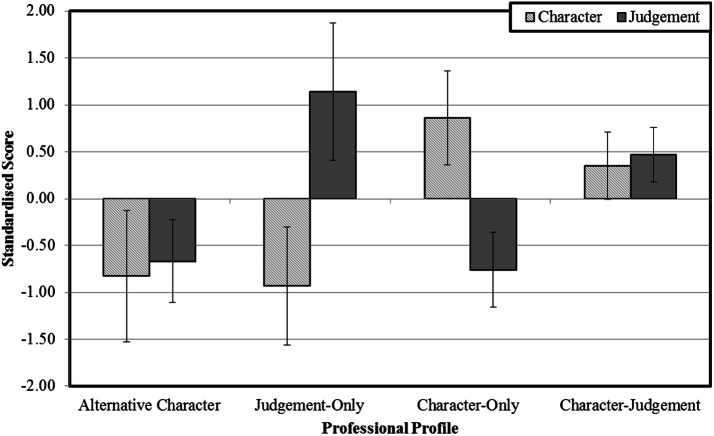
Graphical depiction of the four professional profiles based on standardised mean scores for professionals’ character and professional judgement. The error bars represent the standard errors from the mean for each profile

The four distinct profiles were identified based on the extent to which they differed from the standardised mean in the dimensions of character and judgement (mean differences across the profiles are presented in Table 4). An *alternative*-*character* profile (*n* = 341, 15%) was identified comprising professionals that valued character and judgement below the sample average. This group would have presumably placed higher value on the transcendent or theological qualities not retained in the primary analysis. A *judgement*-*only* profile (*n* = 713, 30%) was characterised by professionals who only valued judgement above the sample average but not character. A *character*-*only* profile (*n *= 964, 41%) reflected professionals who valued qualities of character at the expense of judgement. Finally, a *character*-*judgement* profile (*n* = 322, 14%) included professionals that valued both qualities of character and judgement concurrently (see Fig. 1 for graphical representation).

**Table 4:** Profile differences in character and professional judgement Z-scores with SD’s, F values and effects sizes

Character group	1. Alternative character		2. Judgement-only		3. Character-only		4. Character-judgement		*F*	*η*_*p*_^2^
	Mean	SD	Mean	SD	Mean	SD	Mean	SD		
Virtue category										
Character	− 0.83^2,3,4^	0.70	− 0.93^1,3,4^	0.63	0.86^1,2,4^	0.50	0.35^1,2,3^	0.36	1718.81*	0.69
Judgement	− 0.67^2,3,4^	0.44	1.14^1,3,4^	0.73	− 0.76^1,2,4^	0.40	0.47^1,2,3^	0.29	2156.33*	0.74

Numerical superscripts indicate statistically significant differences (all *p *< .05) between the respective groups for qualities of character and professional judgement, based on Tukey’s honestly significant difference test

*p < .001

Significant differences in the gender distribution across the four professional profiles were found, χ^2^ (3, *n* = 2245) = 55.04, *p* < .001. Forty-seven percent of female professionals were categorised by a character-only profile compared to 35% of men, whereas 25% of females were categorised within the judgement-only profile in contrast to 39% of men. There were also significant differences in the distribution of professionals’ stage of career across the four profiles, χ^2^ (3, *n* = 2281) = 9.42, *p* = .02. The distribution of both cohorts was similar across the judgement-only and character-only profiles (%Δ < 2), with a slightly higher proportion of entry-level professionals located within the alternative-character profile compared to established professionals who had a greater proportion reflecting a character-judgement profile (all %Δ < 4). Additional independent sample *T* tests revealed statistically significant differences in professional purpose across stage of career (*t* = − 15.05, *p* < .001), but not between genders (*t* = .63, *p* = .53)^4^. Specifically, established professionals reported higher senses of professional purpose (standardised mean = 0.33) compared to entry-level professionals (standardised mean = − 0.29). Consequently, stage of career was included as a covariate in all subsequent analyses. The distribution of different professions across the professional profiles was also found to be significant, χ^2^ (12, *n* = 2340) = 136.05, *p* < .001. No substantial differences were found in the distribution of professions across the alternative character (%Δ < 4) and character-judgement (%Δ < 7) profiles. Only 14% of nurses were represented in the judgement-only group, compared to 40% of teachers, 39% of lawyers, 32% of business professionals and 29% of doctors. In contrast, 59% of nurses were categorised with a character-only profile, compared with 43% of doctors, 39% of business professionals, 32% of lawyers and 31% of teachers.

### Character Profile Differences in Professional Purpose

**Fig. 2:**
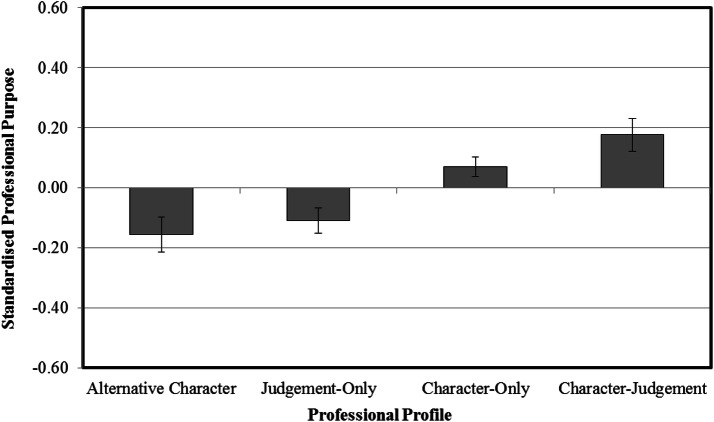
Standardised mean differences in professional purpose across the four professional profiles. The error bars represent the standard errors from the mean for each profile

ANCOVA identified significant differences in professional purpose across the professional profiles when controlling for stage of career, *F*(3, 2054) = 7.92, *p* < .001; *η*_*p*_^2^ = .011. Figure 2 illustrates the standardised mean differences in professional purpose across the four profiles. Post hoc Tukey’s Honestly Significant Difference tests revealed that the character-judgement profile was statistically higher in perceived professional purpose compared to the judgement-only (*p* < .001) and alternative-character (*p* < . 001) profiles, respectively, but not the character-only profile (*p* = .40). The character-only profile reported higher professional purpose than both the alternative-character (*p* < .01) and judgement-only (*p* < .01) profiles. No differences in professional purpose were evident between the alternative-character and judgement-only profiles (*p* = .91). For further inquiry, the testing of an interaction effect revealed that the relationship between the four professional profiles and professional purpose did not meaningfully differ across the five professional domains, *F*(12, 2038) = 1.10, *p* = .35; *η*_*p*_^2^ = .006.

## Discussion

Grounded in virtue ethics and positive psychology, the primary aim of the present study was to take a holistic account of professionals’ valuation of qualities that pertained to character and judgement. Extending literature on character-based judgements (e.g. Crossan et al. 2013; Sturm et al. 2017), professionals were clustered into four distinct profiles depending on the prominence they gave to character and judgement. One profile consisted of practitioners that valued dimensions of character and judgement in balance, two separate profiles comprised professionals that either valued character at the expense of judgement, or vice versa, with a final profile displaying a lack of value for both dimensions. Examination of these profiles revealed that, regardless of stage of career, practitioners valuing character reported greater experiences of ‘good’ professional purpose compared to groups that devalued character. Contrary to prior expectations, these differences were evident regardless of character being valued simultaneously or in isolation of judgement. Philosophical interpretations of these profile distinctions offer new insights into what may constitute a ‘good’ professional and may be informative for future empirical explorations of character in the professional realm. From an applied perspective, the profiling of professionals based on character-judgement may be of substantive worth for regulators in emphasising the importance of balancing character and judgement in the professional domain.

### Character-Judgement Profiles

The profiling of professionals offers a unique opportunity to explore professionals’ character by considering the interplay between their valuation of character and judgement. In line with an Aristotelian conceptualisation of “*phronetic* virtue” (2009, [1142b23–32]), a valuation of character infused with judgement is proposed to epitomise a ‘good’ professional (see Kristjánsson 2015). Judgement forms a central component of true character, offering practitioners a method of adjudicating the relevant dispositions of character that can be drawn upon throughout decision-making processes and deliberation over potential courses of action. Practitioners that give prominence to character without judgement are theorised to display a habituated moral fibre or ‘natural virtue’ but which is not fully *phronesis*-guided (Aristotle 2009, [1142b23–32]). These practitioners may be at risk of inappropriately discerning, and applying, the qualities of character required for specific professional situations. For example, the honesty of a medical professional may constitute a vice if compassion for patients’ feelings is not also judged to be necessary. On the contrary, professionals that value judgement without the internal excellences of character may be prone to use judgement in a narrowly instrumental or even vice-like manner for self-serving motives, rather than for the benefit of society or their organisation (Moore 2015).

The present findings indicate that practitioners valuing character may be more likely to report higher levels of a ‘good’ purpose for their profession, yet unexpectedly an explicit value of judgement was not found to be essential. One plausible interpretation for this finding may be that character, or more specifically the virtues underpinning character, are fundamental to the development of a wider societal purpose (see Moss 2011). Aristotle proposes that virtues are principally important insofar as they direct an individual towards a right and ‘good’ end or goal (Aristotle 2009 [1144a7–9, 1145a1–5]). Conversely, judgement in the *phronesis* sense is more concerned with balancing the means of these virtues which, in turn, facilitates an individual to actualise this end purpose. The present self-reported measure of purpose taps into professionals’ identification with a ‘good’ purpose, as opposed to how their actual behaviour aligns with it, and thus the character criterion is likely to yield greater influence. Professional purpose as reflected by a volitional striving to do useful work for the betterment of society has close connotations with character virtues pertaining to humanity (Peterson and Seligman 2004), sociability (Shryack et al. 2010), conscientiousness (Macdonald et al. 2008) and emotional care for others (McGrath 2015). Collectively, these dimensions of character are symbolic of civic virtues which, when valued, may direct professionals to a purpose associated with citizenship and social responsibility (see Garofalo and Geuras 2005). Thus, in accord with MacIntyre (1981), practitioners that put personal preference towards internal qualities of character, such as fairness or kindness, may be more likely to identify with the ethical dimensions of their practice, more so than practitioners who give less prominence to these excellences of character.

The finding that the character-judgement and character-only profiles did not differ in professional purpose offers further insights into what may constitute a *phronimos* professional. A possible explanation may be that the two groups, in fact, reflect equivalent dispositions for *phronesis*-informed character. It is conceivable that practitioners with a character-only profile may predominantly place importance upon qualities of character, but could do so with an implicit endorsement of sound judgement when they employ these qualities. In this case, these character-only practitioners may actually demonstrate what is known as ‘complete virtue’ whereby the value of character becomes intrinsically attached to judgement and deliberation (Aristotle 2009 [1142b1–36, 1143a1–37, 1143b1–16]). In contrast, practitioners with a character-judgement profile may more accurately embody Macintyre’s portrayal of *praxis* (1981), encompassing a similar state of *phronetic* virtue as the character-only profile but with their additional value of judgement potentially reflecting external competencies which are instrumental for good profession-specific practice. The value of the judgement component may, therefore, reflect an external intellectual complement to the virtues which could be indicative of ‘cleverness’ as opposed to character-led deliberation synonymous with ‘*phronesis*’ (see Aristotle 2009, [1144a23–31]). Hence, it may be the value of character in the present findings, rather than judgement, which is indicative of the *phronimos* professional. These ‘character’ professionals may have internalised the ‘good’ through the intrinsic virtues themselves which are accompanied by the implicit judgement to critically evaluate when and how to employ these ‘good’ qualities of character (Kinsella and Pitman 2012).

The aforementioned considerations highlight the empirical challenge of accurately measuring judgement, qua *phronesis*, as balancing the means of competing virtues. An issue with contemporary assessments of judgement is that they tap into intellectual qualities which reflect being cognitively complex, imaginative or analytical. For example, the component of *wisdom* in the VIA includes qualities such as creativity, curiosity and love of learning (Peterson and Seligman 2004). These qualities are not necessarily essential for deliberating how to balance the means of different virtues but rather reflect intellectual qualities in themselves which are concerned with a search for knowledge. Component analyses consistently cluster these intellectual qualities into factors reflecting cognitive strengths or inquisitiveness towards an environment (McGrath 2015; Peterson et al. 2008; Ruch et al. 2010). In contrast, judgement as indicative of Aristotelian *phronesis* is symbolised by situational awareness, reflective perspective-taking and critical thinking (see Crossan et al. 2017; Darnell et al. 2019). Alternative measurement methods to the VIA may be needed to finely assess the interplay between the key philosophical propositions of *phronesis* and distinct dimensions of character. Future studies could also extend the current profiling of professionals to explore how these groups may differ in their professional efficiency and actual decision-making. Although both ‘character’ groups report similar levels of purpose, it would be worthwhile determining whether professionals are more proficient in their practice when simply applying *phronesis* spontaneously (i.e. character-only profile) or when *phronesis* may be accompanied by conscious attention towards extrinsic competencies that could be relevant for ‘good’ practice (i.e. character-judgement profile).

Nevertheless, practitioners portraying a judgement-only profile in the present study seem to overweight the importance of judgement at the expense of qualities of character. As hypothesised, the findings indicate that these practitioners may be more likely to value judgement to seek ends which are not aligned with the wider purpose that their practice is intended to serve (MacIntyre 1981; Moore 2015). Consequently, when judgement is not synergised with dimensions of character, it may be directed in a vice-like manner towards self-serving or vicious ends (Aristotle 2009, [1144a23–31]). These practitioners depict the very profile that MacIntyre cautions against as they may be less likely to draw upon qualities of character to guide their decision-making when dealing with changeable professional scenarios (Carr 2018; Seijts et al. 2015). The realisation of this judgement-only profile offers a basis for further research to explore whether professionals with such a profile may be more prone to incidents of systematic malpractice and professional misconduct. Inferences from previous work (e.g. Duffy et al. 2011; 2012) suggest that the evidenced lack of volitional purpose by this judgement-only group may put these practitioners at greater risk of becoming less committed and accountable for their actions which may bring about asocial or amoral consequences.

It is notable that the valuation of judgement alone, without character, was found not to yield any greater sense of professional purpose than when neither judgement nor character was valued (i.e. the so-called alternative-character profile). It should be accentuated that practitioners displaying an alternative-character profile in the present study do not lack character per se, but likely place value upon qualities which reflect transcendent and theological dimensions of character (e.g. love, spirituality, hope; McGrath 2014; Ruch et al. 2010). Such qualities have been found less relevant for organisational contexts and outputs (e.g. Crossan et al. 2017), but are closely associated with personal well-being and life satisfaction (e.g. Feldman & Snyder 2005; Peterson et al. 2009; Wood et al. 2011). It is possible that these alternative-character practitioners experience high levels of general well-being, but their personal values are out of sync with qualities promoted within normative professional cultures and working environments, which inhibit their sense of professional purpose. In contrast, groups of practitioners deemed to value character in the present study may actually represent those whose personal values are more aligned with the qualities rewarded or facilitated in day-to-day professional environments (see Moore 2015, 2017). These latter practitioners may have experienced a process of ‘sensitisation’ in which their personal character has become more attuned to the requirements and normative working cultures they encounter in their workplace (e.g. Beadle and Moore 2006). Further examination of professional profiles with regard to those that ‘feel good’ compared to those that may ‘do good practice’ in their professional role might help to illuminate these aspects. For instance, it may be that practitioners with an alternative-character type are less effective in their role than their colleagues. Alternatively, it may be that the transcendent and theological qualities that these professionals value are overlooked in professional environments or are thwarted by workplace constraints such as lack of time or assessment pressures. Organisational leaders may be best advised to consider the character qualities that are fostered and promoted throughout the daily operations of their organisation, or wider profession (e.g. Seijts et al. 2019).

### Practical Implications of Findings

In addition to the theoretical implications, professional regulators would be well advised to consider the current profiling analytical approach in recognising practitioners that may under-value or over-value aspects of character and judgement. This analysis method could have valuable insights for pre-service training to ensure future professionals develop an understanding of ‘good’ character as they gain early experiences which inform their professional judgement. In light of incidents of professional malpractice and poor professional judgement, it may be beneficial for continuing professional development (CPD) programmes and work environments to be grounded upon an impetus towards character-based judgements (e.g. Mulvey 2013; Rest and Narváez 1994). This may be especially important for practitioners overweighting the importance of judgement at the expense of character. Character can be embedded in organisations, and their practitioners, when given explicit attention through targeted strategies that promote ‘good’ character (see Leader Character Insight Assessment; LCIA; Furlong et al. 2017; Seijts et al. 2017). Such strategies include reorienting performance management processes to provide more constructive feedback using a clear language of character (e.g. Crossan et al. 2017), assigning professionals with regular training activities to consider how character may inform their decision-making (e.g. ethical dilemmas; Dutelle and Taylor 2017), and behavioural modelling from professional leaders that demonstrate and nurture well-informed character in their colleagues (e.g. moral exemplars; Carr 2018). It is also important to qualify that the effective implementation of such initiatives may require alleviation of other pressures currently encountered within many professional contexts, such as excessive auditing, rigorous assessments and budgetary constraints, which may present obstacles to the cultivation of character in the workplace (Evetts 2009).

In addition to exploring the professional domain at a general level, consideration of these professional profiles may be of substantive benefit for regulators within distinct professional fields. For instance, in accord with previous evidence (Peterson et al. 2010), a large proportion of nurses portrayed a character-only profile with less importance given to judgement. By its nature, the nursing profession is underpinned by a concern for the ‘ethics of care’, with dimensions such as humanity, compassion and integrity featuring prominently in patient expectations of nurses (Swanson 1993). While this emphasis on character is essential to the profession, regulators overseeing training and guidance in the nursing profession might also seek to ensure that nurses’ decision-making is informed by sound autonomous judgement, rather than an overreliance on strict codes of conduct (Grace 2017). In contrast, a higher proportion of professionals in teaching and law displayed a judgement-only profile. Teaching has traditionally been viewed as a vocation with a *prima facie* commitment to facilitate the education and personal development of others (Carr 2011). Recent shifts in UK education policies, however, have imposed a culture of targets and assessment pressures which may cause teachers to prioritise more instrumental and performance-related ends (Edgington 2016). Evidence would suggest this culture change may have negative connotations for teachers’ well-being and job persistence (e.g. Kidger et al. 2016). In the face of these pressures, the task for educational bodies to ensure that teachers and education leaders do not lose sight of the moral duty that underpins their professional practice becomes more pressing (Sanger and Osguthorpe 2015). Similarly, the role of legal professionals is to uphold the law of a just and fair society, yet the demands of clients and emphasis on profit margins, certainly in large law firms, appear more synonymous with private sector professions (e.g. Feenan et al. 2016; Furlong et al. 2017). It remains important that legal regulators ensure that working cultures in law firms emphasise the ethical and social objectives of lawyers so that their independent judgement is synergised with qualities of ‘good’ character. Although the present findings emphasise individual character profiles, the bespoke and inherent requirements within specific professional cultures remain an essential consideration.

## Limitations and Future Research Directions

The current research offers a starting point for wider empirical exploration regarding professionals’ character-infused judgement and the implications for their professional service. The present work specifically focuses on how professionals’ character profile may correspond with a sense of professional purpose. Nevertheless, a wider societal purpose for professional work is only one component of a ‘good’ professional and does not equate to appropriate professional action and decision-making. Future research could adopt the present profiling technique to consider how practitioners with different character compositions respond to situations in their choice of action and their reasoning for this action. In accord with the philosophical components of *phronesis*, further research may help ascertain if practitioners of character-informed judgement may practice in a more appropriate manner with greater moral motivation and reasoning (see Bebeau and Thoma 2013). In addition, the present assessment of professional purpose was conducted through a single measure, deriving from previously used items. Future investigations of professional purpose may explore if the patterns found in the present work are replicated when using multiple measures of meaning and purpose at work, such as the Work as Meaning Inventory (Steger et al. 2012) and the Work Volition Scale (Duffy et al. 2012). Utilising multiple measures may help provide a more comprehensive assessment of practitioners’ purpose for their profession.

Character and judgement were assessed through hierarchal rankings in the current work which offers insights into the dimensions practitioners gave particular importance to. Practitioners’ values may be reflective of their character disposition, yet they do not depict the extent that these practitioners assume or exercise these dimensions in their working conduct. Replicating the current profiling procedure using quantitative scores of character and judgement would help identify the extent to which professionals may actually endorse, or lack, each dimension in their professional role. Doing so would enable the concept of character to be explored more precisely by assessing if all distinct dimensions of character (e.g. humanity, justice, courage, temperance) are endorsed simultaneously and synergised with sound judgement (Schwartz and Sharpe 2006). Furthermore, such an investigation may reveal specific reasons for the lack of difference in professional purpose between the character-only and character-judgement profiles. Namely, do both groups valuing the character virtues utilise implicit sound judgement when they operationalise these virtues? Finally, the cross-sectional design of the present research prevents temporal associations from being examined. Additional longitudinal research may explore how variations in professionals’ character profiles may associate with changes in their perceived professional purpose over time, and allow within- and between-profile associations to be examined across multiple years of practice.

## Conclusion

Contemporary professional culture has increasingly imposed constraints on practitioners’ agency, often resulting in the prioritisation of commercial or materialistic objectives at the expense of a focus towards ‘good’ character (Carr 2018; Moore 2015). The present findings lend credence to the important role that a value of character can have in the pursuit of ‘good’ purposeful practitioners. In accordance with philosophical propositions (e.g. MacIntyre 1981), practitioners that do not give prominence to internal excellences of character, even when giving importance to their own judgement, may be at risk of disconnection from the wider purpose that the professions are broadly intended to serve. Conceptually, the findings open the door for further empirical exploration of what may constitute a *phronimos* professional (Aristotle 2009), and pave the way for new research to further explore the interaction between character and judgement in professional spheres. From a practical perspective, practitioners’ character composition should be a central component of discussions surrounding professional competency, and not a subset of practitioners’ professional responsibilities (Seijts et al. 2019). Professional organisations should be aware of their members’ character disposition and ensure that initiatives are put in place to foster their value for character and judgement upon entering, and throughout, their professional career.

## Footnotes

1 The “character strengths” posited within positive psychology (Peterson and Seligman 2004) are conceptually equivalent to Aristotelian “virtues” (Aristotle 2009). The fundamental distinction is that positive psychology refers to the amount of experience in specific strengths, whereas Aristotle advocates the mean experience of virtues are better rather than the overall quantity (Kristjánsson 2015; Schwartz and Sharpe 2006).

2 Also see similar work regarding the links between dimensions of character with organisational citizenship and work-identity (Hur et al. 2016; Rego et al. 2010).

3 All data was collected by the Jubilee Centre for Character and Virtues, University of Birmingham.

4 Binary dummy codes were used to distinguish between stage of career (0 = entry-level; 1 = established professionals) and genders (1 = female; 2 = male).
